# Enhanced expiratory rebreathing space for high loop gain sleep apnea treatment

**DOI:** 10.3389/frsle.2023.1248371

**Published:** 2023-09-29

**Authors:** Thomas Quinn, Robert Joseph Thomas, Eric James Heckman

**Affiliations:** Department of Medicine, Division of Pulmonary, Critical Care and Sleep Medicine, Beth Israel Deaconess Medical Center, Boston, MA, United States

**Keywords:** complex sleep apnea, loop gain, periodic breathing, EERS, carbon dioxide, dead space

## Abstract

The pathophysiology of sleep apnea goes beyond anatomic predisposition to airway collapse and includes additional factors such as arousal threshold and loop gain. High loop gain is a prominent feature in central and complex sleep apnea (with a mixture of obstructive and central features) where relative hypocapnia can lead to respiratory instability and periodic breathing. Existing therapies, including continuous positive airway pressure (CPAP) and adaptive servo-ventilators, often inadequately treat sleep apnea with high loop gain features. Enhanced expiratory rebreathing space (EERS) targets prevention of the hypocapnia that triggers central events in sleep by increasing dead space in amounts less than typical tidal volumes. This is accomplished by covering traditional exhalation ports on positive airway pressure masks and adding small additional tubing with distal exhalation and safety valves. This technique reduces carbon dioxide (CO_2_) blow-off during arousals and the associated large recovery breaths, typically producing a maximal increase in resting CO_2_ by 1–2 mmHg, thus increasing the CO_2_ reserve and making it less likely to encounter the hypocapnic apneic threshold. Typically, the amount of EERS is titrated in response to central events and periodic breathing rather than aiming for a goal CO_2_ level. Ideally CO_2_ monitoring is used during titration of EERS and the technique is avoided in the setting of baseline hypercapnia. This method has been used in clinical practice at our sleep center for over 15 years, and retrospective data suggests an excellent safety profile and high rates of successful therapy including in patients who have previously failed CPAP therapy. Limitations include decreased effectiveness in the setting of leak and decreased tolerance of the bulkier circuit. EERS represents a simple, affordable modification of existing positive airway pressure modalities for treatment of central and complex sleep apnea. Areas of future study include randomized controlled trials of the technique and study of use of EERS in combination with adaptive ventilation, and pharmacologic adjuncts targeting high loop gain physiology.

## Introduction

It is now generally accepted that the pathophysiology of obstructive sleep apnea (OSA) involves non-anatomical traits, including high loop gain, an impaired negative pressure response, low arousal threshold, increased arousal intensity and sleep fragmentation. However, clinical guidelines and management of apnea has largely ignored the growing research data supporting the importance of considering an endotype/phenotype driven approach to optimized and personalized sleep apnea care. In those with hypocapnic central sleep apnea (CSA), there is little argument that high loop gain and hypocapnia is a key destabilizer of sleep-respiration (Javaheri and Badr, 2023). The hypercapnic ventilatory response is in fact elevated in idiopathic CSA (Xie et al., 1994, 1995). In those with OSA, high loop gain will result in a tendency to hypocapnia. Yet, measurement or manipulation of carbon dioxide (CO_2_) has largely remained confined to research laboratories. Here, we present the logic behind, the practical application of, and our results with the use of dead space plus positive airway pressure, a method we call Enhanced Expiratory Rebreathing Space (EERS).

## Sleep apnea phenotypes and control of respiration—the importance of CO_2_

There is increasing appreciation of the varied phenotypes of sleep apnea (Malhotra et al., 2020; McNicholas and Pevernagie, 2022). Although OSA, with an anatomic predisposition to airway collapse is the most commonly invoked mechanism, it has long been considered only a partial contributor (Remmers et al., 1978; Mezzanotte et al., 1996; Younes et al., 2007). While other factors such as failure of muscle recruitment/compensation can contribute to OSA (Younes et al., 2012, 2014), these factors fail to explain sleep apnea of a central nature, or, the common variant of a mixture of both obstructive and central disease (complex sleep apnea) (Gilmartin et al., 2005). Although the establishment of the diagnosis of treatment-emergent sleep apnea (TE-CSA) has raised awareness of pathophysiological “mixed” sleep apnea (ICSD-3), it oversimplifies the patterns of sleep apnea to an artificial “all or none” format where one physiology clearly dominates. In fact, TE-CSA merely reflects the consequence of targeting only the upper airway when breathing control instability is also present. In reality, the features of reduced respiratory effort and obstruction are often intertwined and evident to some extent across both diagnostic and titration sleep studies. Central events, with prolonged exhalation and reduced airway stenting can produce airway collapse and obstruction (Badr et al., 1995). Given this, the label complex sleep apnea may be more appropriate (Gilmartin et al., 2005; Morgenthaler et al., 2006). Thus, multiple pathophysiologic factors contribute to these different phenotypes, including a low arousal threshold and loop gain (Table 1) (Eckert et al., 2013).

**Table 1:** Relative contribution of pathologic features in different sleep apnea endotypes.

**Sleep apnea type**	**Airway narrowing**	**Loop gain**	**Arousal threshold**	**Role for EERS**
Obstructive	Significant	Average	Variable	No
Central	Minimal	High	Variable	Yes
Mixed	Significant	High	Low	Yes
Obesity hypoventilation syndrome	Mild to significant	Low	High	No

The sleep-related arousal threshold describes the ease with which an individual can be triggered to arouse from sleep, with a low arousal threshold suggesting that even mild stressors (respiratory or non-respiratory) can lead to an arousal. Conversely, a high arousal threshold suggests that a high amplitude stressor is required to disrupt sleep. In the context of sleep apnea, a low threshold will lead to more frequent sleep wake transitions (Jordan et al., 2017) and seems to have consequences for tolerance of positive pressure therapy (Zinchuk et al., 2021). Targeting this factor underlies the principle of using sedatives to treat sleep apnea (Eckert et al., 2011; Edwards et al., 2016; Ahmad et al., 2023). However, trials examining monotherapy with sedatives to control sleep apnea have been inconsistent (Rosenberg et al., 2007; Carter et al., 2018). A high arousal threshold is a predictor of a positive response to hypoglossal nerve stimulation for sleep apnea, consistent with a role for the arousal threshold in modulating outcomes of sleep apnea (Op de Beeck et al., 2021).

Loop gain refers to the relation of a response to a disturbance, for sleep apnea the ratio of ventilatory response in reaction to a ventilatory stimulus. When the loop gain is higher than desirable, there is a disproportionately robust ventilatory response, and when lower than desirable, and over-damped system. A vigorous ventilatory response may seem advantageous compared to the alternative, a low loop gain leading to an insufficient ventilatory response, and hence a tendency to hypoventilation. However, high loop gain causes its own challenges given the control mechanisms governing respiration in sleep (Younes et al., 2001). Loop gain can be further classified as controller gain, mixing gain and plant gain. Controller gain refers to the sensitivity of the system to changes in chemical stimuli like carbon dioxide and is governed by central and peripheral chemoreceptors (Orr et al., 2017; Roberts et al., 2022). High controller gain suggests that a given change in PaCO_2_ will result in a greater change in ventilation, while a low controller gain will generate a lesser response in ventilation for the same change in PaCO_2_. In sleep apnea, intermittent nocturnal hypoxia sensitizes the carotid body and results in a steeper slope of the hypoxic ventilatory response and elevated controller gain (Tamisier et al., 2009). Plant gain refers to the efficiency of gas exchange within the respiratory system; it is dependent on the characteristics of the individual's cardiopulmonary systems. An individual without cardiopulmonary comorbidities will have a higher percentage of their lung volume participating in efficient gas exchange. A normal pulmonary system leads to more change in gas levels per change in minute ventilation than an individual with, for example, advanced chronic obstructive lung disease (COPD). The COPD patient has lower plant gain and is less likely to produce a large change in CO_2_ for a given change in ventilation. In a system with high loop gain, a low efficiency of gas exchange can actually help prevent hypocapnia and hence the resultant overshooting of the ventilatory response. In addition, an “arousal gain” can be considered a modifier, with more vigorous arousals a result of greater effective controller gain. Mixing gain is most relevant in conditions like heart failure with a prolonged circulation time.

The control of ventilation is quite different in the states of wake and sleep. Inputs are much more numerous during wakefulness and include the peripheral and central chemoreceptor response to multiple molecules including oxygen, hydrogen ions, CO_2_, nitric oxide and hydrogen sulfide; input from temperature and pain stimuli; lung stretch; emotional stimuli; and voluntary control of breathing (Del Negro et al., 2018). The influence of most of these inputs wanes with a transition to sleep. In the sleep state, voluntary and emotional stimuli are absent and respiration during non-rapid eye movement (NREM) sleep is largely governed by chemical drivers. Specifically, CO_2_ becomes the key respiratory driver, such that hypercapnic respiratory response is the greatest determinant of ventilatory drive during sleep. The NREM CO_2_ threshold is just a few mmHg lower than CO_2_ values under eupnea. The CO_2_ reserve, therefore, is the space where CO_2_ may fluctuate without triggering ventilatory instability. CO_2_ reserve is low in those with hypocapnic CSA, periodic breathing, or TE-CSA (Dempsey et al., 2004; Xie et al., 2011, 2013). The ventilatory response to hypercapnia and hypoxia are also more robust during wake as compared to sleep, with more substantial increases in ventilation in response to CO_2_ increase or O_2_ decrease.

The changing inputs to respiration upon sleep onset result in an inherently unstable state. The loss of behavioral control, reduced respiratory drive, and reduced chemosensitivity make hypoventilation more likely, while decreased muscle tone increases obstruction; as a result, there is an increased risk for flow limitation. The mild retention of CO_2_ after sleep onset offers some protection for respiratory stability as it increases the PETCO_2_-PCO_2_ apneic threshold difference. Progressive flow limitation causes a reduction in ventilation and a proportionate response dictated by the individuals' loop gain. In the situation of high loop gain, a robust respiratory response to this reduction in ventilation increases the risk of inducing hypocapnia below the hypocapnic apneic threshold and thus induction of a central apnea. A resulting apnea subsequently leads to CO_2_ re-accumulation and therefore increased ventilatory drive, which is disproportionately high in the setting of high loop gain, generating an alternating cycle of relative hypo- and hypercapnia. Ultimately, this cycle can cascade to the point that periodic breathing is generated and maintained. The combination of both high loop gain and low arousal threshold can be particularly problematic, as the tendency to quickly arouse increases the frequency of transitions between wake and sleep, allowing for significant fluctuations in ventilatory drive and therefore the risk of over-ventilation in the setting of high loop gain. Even in patients with primary OSA, objective measurement of arousal threshold and loop gain suggest that about a third of patients have high loop gain and a third have low arousal threshold; this suggests that these factors are relevant across all forms of sleep apnea (Eckert et al., 2013). Although formal measurement of loop gain and arousal threshold is not typically utilized in clinical practice, features collected by typical polysomnography can hint at these characteristics (Table 2).

**Table 2:** Polysomnographic features suggestive of high loop gain and low arousal threshold.

**Features suggestive of high loop gain**	**Description**
NREM dominance of events	•Events cluster around sleep wake transitions when overventilation has maximal impact at inducing central events •Respiratory events markedly improved in REM vs. NREM sleep, ideally a supine-to-supine comparison
Periodic breathing	Cyclic under-shoot and over-shoot of ventilation creating a metronomic self-similar pattern
Oximetry banding	Series of self-similar events of consistent duration and oxygen desaturations produces a thick line on oximetry when viewed on the scale of the entire night
Treatment emergent central apneas	Increased ventilation triggers central events in the setting of low CO_2_ reserve
Prolonged time between event onset and oxygen nadir associated with the event	Cardiac insufficiency leads to a mixing delay, an increased transit time of relatively deoxygenated blood to reach the peripherally located oximetry sensors
**Features suggestive of low arousal threshold**	**Description**
Elevated Spontaneous Arousal Index	Suggestive of easy arousal
Non-hypoxic sleep apnea	AHI3% significantly greater than AHI4%, suggestive fragmentation is not driven by gas exchange abnormalities
Elevated N1 sleep	Evidence of frequent sleep stage transitions
Persistent cyclic alternating pattern (CAP) of EEG despite optimal upper airway support	Indicative of frequent EEG arousals and arousability

Central apneas induced by relative hypocapnia during sleep are a well-established phenomenon. In the intubated and sedated ICU patient, alkalemia induced during a control mode of mechanical ventilation will result in apneas upon the transition to pressure support modes due to low levels of CO_2_ and subsequent lack of respiratory drive. At high altitude, the reduced FiO_2_ leads to increased minute ventilation based on the hypoxic respiratory response. This increased minute ventilation leads to hypocapnia below that achieved at sea level and thus central apneas and high attitude periodic breathing result (Masuyama et al., 1989; Khoo et al., 1996; Fowler and Kalamangalam, 2002; Lombardi et al., 2013; Pramsohler et al., 2019). In TE-CSA, the appropriate improvement in ventilation with treatment of obstruction leads to relative hypocapnia and induction of respiratory instability (“high altitude at sea level”). Patients with high loop gain, such as those with congestive heart failure and idiopathic CSA, lack the typical degree of hypoventilation with transition to sleep and the ventilatory response to CO_2_ below eupneic values is more sensitive. This combination makes it more likely for these high loop gain CHF patients to reach the hypocapnic induced apneic threshold (Xie et al., 2002).

Despite the fact that central events induced by relative hypocapnia are a common feature of sleep breathing, traditional treatments of sleep apnea remain insufficient to treat sleep apnea phenotypes enriched for central events, whether due to primary CSA, or complex sleep apnea with a mixture of central and obstructive events. The CANPAP trial showed that continuous positive airway pressure (CPAP) leaves a substantial residual burden of central apneas in patients with congestive heart failure and fails to improve heart failure related outcomes (Bradley et al., 2005). Adaptive servo ventilators are designed for Hunter-Cheyne-Stokes respiration, a classic feature of high loop gain. These bilevel machines are designed to adjust the pressure delivered relative to respiratory effort (inverse, anti-cyclic) in attempts to break the cycle of periodic breathing. Practically, the ability of these proprietary algorithms to successfully prevent periodic breathing may be brand-specific, as different models produce substantially different minute ventilations in the same individual (Knitter et al., 2019). Pathological pressure cycling imposes hemodynamic stress on the cardiovascular system (Gunn et al., 2018). Although studies suggest that adaptive servo ventilators are superior to CPAP for treatment of the respiratory features of complex sleep apnea, the impact on sleep quality and clinical outcomes is less striking (Morgenthaler et al., 2014). The expense of these machines and strict coverage criteria are challenges (Morgenthaler et al., 2021). The SERVE-HF trial demonstrated increased mortality in those with systolic heart failure, which severely undercuts the utility of the device in those patients at elevated risk for high loop gain (Cowie et al., 2015). Acetazolamide (discussed in more detail below) shifts the CO_2_ response curve to the left and lowers the apneic threshold, improving respiratory stability. Supplemental oxygen has been demonstrated to have a positive effect on sleep apnea with high loop gain (Sands et al., 2018). However, oxygen monotherapy cannot overcome upper airway resistance. In addition, it can be difficult to qualify for in the context in the US insurance system, and self-pay has a significant long-term expense world-wide (Morgenthaler et al., 2021).

## Introducing dead space/enhanced expiratory rebreathing space

Based on the physiology of hypocapnic induced central apneas, an intervention preventing hypocapnia would be a logical target. CO_2_ modulation has been utilized as a strategy to stabilize periodic breathing and other chemoreceptor-associated breathing abnormalities for over 40 years. Berssenbrugge and colleagues first demonstrated in 1983 that hypoxia-induced periodic breathing could be eliminated by augmenting inhaled FiCO_2_, resulting in a 3–6 torr increase in arterial PaCO_2_ and normalization of breathing pattern, supporting the hypothesis that periodic breathing is due in part to transient oscillations in arterial CO_2_ content above and below the CO_2_ apnea threshold (Berssenbrugge et al., 1983). Dead space has been used to improve sleep-breathing and thus sleep in mechanically ventilated patients (Parthasarathy and Tobin, 2002), periodic breathing at high altitude (Lovis et al., 2012; Patz et al., 2013), idiopathic CSA (Xie et al., 1997), and heart failure (Khayat et al., 2003). The “dose” required to stabilize ventilation may not meaningfully improve sleep quality or arousals (Szollosi et al., 2004).

The first demonstration of the benefits of a low concentration of CO_2_ that was “clamped” to just above the NREM sleep CO_2_ threshold was in 2005 (Thomas et al., 2005). In that report, using an investigational device, a concentration of 0.5–0.8 (% CO_2_) was sufficient to enable respiratory stability when combined with positive pressure airway support. This realization motivated the trial of a small amount of dead space (50–100 cc, vs. the 300–500 cc used in prior reports) with CPAP. The concept of Enhanced Expiratory Rebreathing Space (EERS) is the adaptation of dead space to concomitant use of positive pressure ventilation (Gilmartin et al., 2010). The EERS space is analogous to the “dead-space” in the native respiratory system, which accounts for the volume of air in each breath that does not interface with the gas-exchanging tissues of the lung (i.e., the volume of air contained within the conducting airways such as the mouth, trachea and bronchi). In the normal adult, anatomical dead space accounts for roughly 33% of the total tidal volume of inspired air (roughly 130–180 cc's per breath) and can be measured more accurately via the Fowler method, or single-breath nitrogen washout test. Other forms of dead space include alveolar dead space which is often secondary to disease (e.g., atelectasis, impaired pulmonary blood flow, or increased alveolar pressure) as well as apparatus dead space from respiratory equipment, such as that utilized in EERS circuitry.

## Biological effects of EERS

EERS has a few possible effects to enable respiratory stability. First, by using EERS, loop gain is lowered through reduction in plant gain, by reducing the efficiency of CO_2_ removal with ventilation. Second, by slightly raising baseline CO_2_ (1–2 mm Hg), the likelihood of hitting the NREM CO_2_ threshold is reduced, and the CO_2_ reserve is therefore increased. Third, the greatest effect of EERS may be during intermittent arousal-induced ventilatory blow-off (a “shock-absorber” effect), preventing the major resulting fluctuations of CO_2_ that inevitably occur. Finally, there may be effects at the level of cerebral blood flow. There is no change in mean heart rate or respiratory rate (Gilmartin et al., 2010).

## Creating EERS

**Figure 1:**
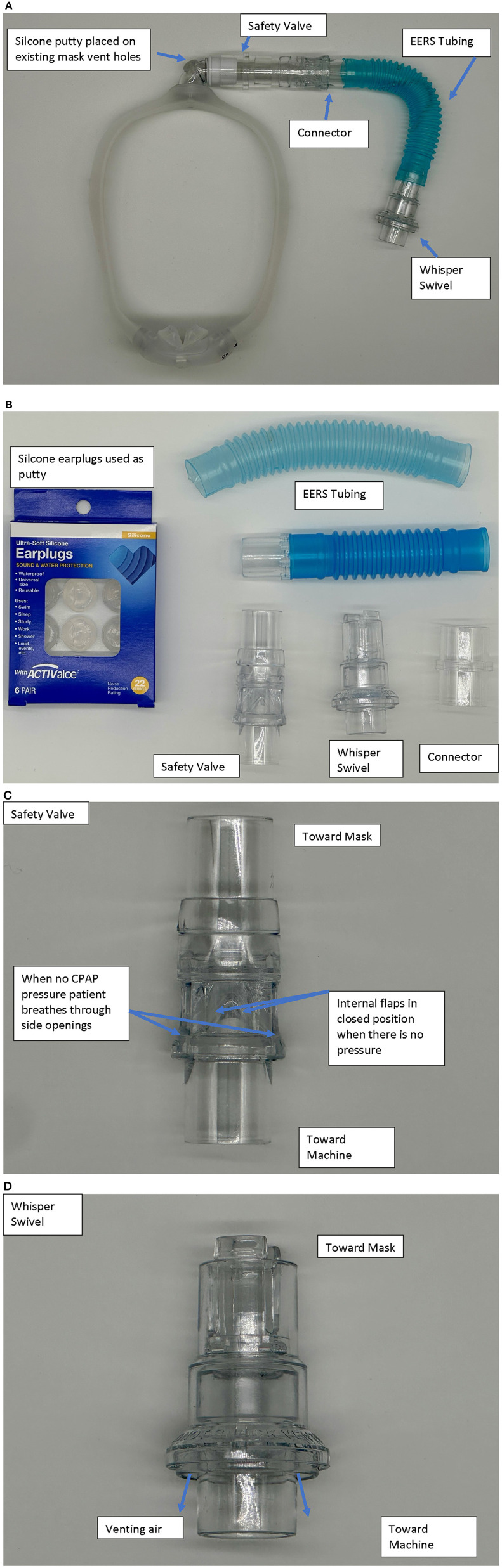
**(A)** Example of a nasal pillow mask set up with the nonvented, EERS modification. The model is a Dreamwear by Respironics-Phillips. **(B)** Description of parts used. Silicone ear plugs (typically CVS Health, or Mack's brand) are utilized to cover the standard mask vents. Other components—Whisper Swivel−332113—Respironics-Philips, Non-Rebreather Valve—NV-HC209—Fisher & Paykel, EERS tubing—PES-1680-(50,100,150) cc—Teleflex/Hudson, Flex Tube with 22 mm connector–PMS-6107—Portex, 22 mm Connectors—HUD1421—Teleflex/Hudson. The sequence is mask-safety valve (if nasal mask)-EERS-Whisper-standard CPAP (with connectors as needed). The connector and extra tubing allow both to connect to typical CPAP tubing and modulation of the amount of EERS. **(C)** The safety valve has a one wave valve that allows air to be entrained through side ports in the event of power loss with discontinuation of positive airway pressure. An alternative is the Hans-Rudolph non-rebreathing valve part numbers 115402 or 115401. **(D)** The Whisper valve serves as the exhalation vent in the series.

**Figure 2:**
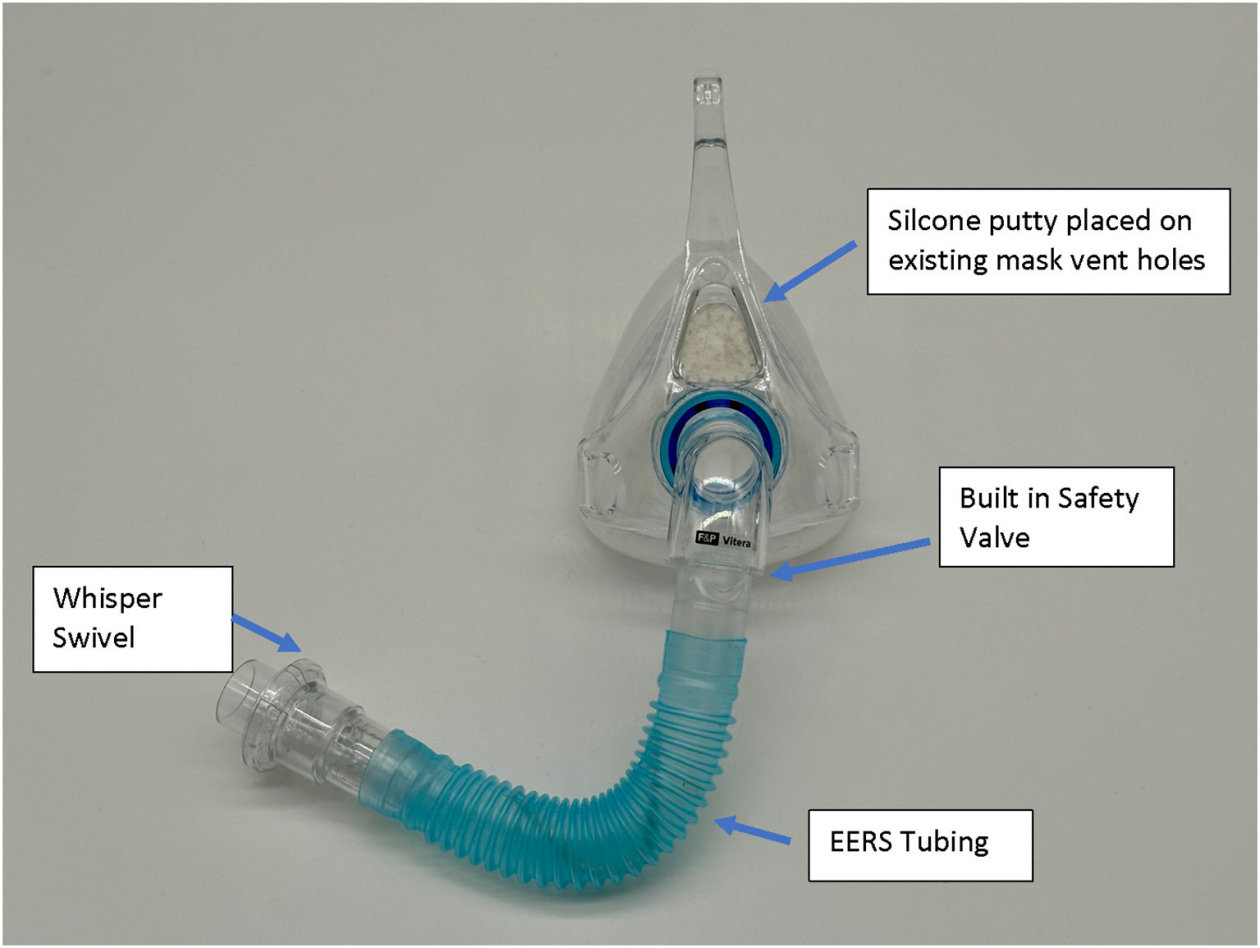
Example of a full-face mask set up with the nonvented, EERS modification. The model is a Fisher & Paykel, F&P Vitera™. The existing internal mask safety valve is utilized with the addition of connectors, EERS tubing, and Whisper Swivel Valve.

Native non-vented masks may be used, or “conversion” of a standard vented mask. It should be noted that some masks like the ResMed AirFit™ N20 has a non-vented configuration with a short stalk. EERS is achieved first by blocking the typical mask exhaust vents, for example by adding a compound such as silicone putty to block the vent holes and prevent normal CO_2_ escape. This step converts a standard “vented” CPAP mask into a “non-vented” mask setup, and adds about 70 (nasal mask) to 100 (oronasal mask) cc's of dead space to the system. Additional EERS volumes are then added by inserting corrugated flexible tubing in 50 cc increments to the mask tubing, with the ability to add 50–150cc's total of EERS to the system. A swivel valve (we use the Philips Whisper Swivel II Exhalation Valves), which allows for continuous venting and thus represents the termination of the non-vented circuit, is added at the distal end of the EERS tubing and allows for exhalation of CO_2_ (Figures 1, 2). In full-face mask setups, a safety valve (non-rebreathing valve) is added to prevent theoretical asphyxiation in the event of a power outage or machine malfunction, but could be considered optional in nasal-only. Most if not all current full-face masks come with an inbuilt non-rebreathing valve, and no added safety valve is required; as a default we use safety valves in nasal masks also. A patient with normal dexterity and mental status could also easily remove the mask in the event of power failure.

## Patient selection

Adherence to positive pressure therapies is a significant predictor for symptomatic improvements in OSA, with usage >6 h nightly associated with decreased sleepiness, improvement in daily functioning, and normalization objective memory performance (Zimmerman et al., 2006; Weaver et al., 2007). In patients with high loop gain sleep apnea, there are some data that non-adherence rates are even higher than those with straightforward OSA (Ni and Thomas, 2023). Measurements of baseline unstable ventilatory control (i.e., high loop gain) have also been associated with elevated residual AHI and rates of CPAP non-response defined as residual AHI >5/h despite adequate control of obstruction, even when CPAP compliance is maintained. This implicates that the measurement of loop gain is a potential *a priori* predictor of patients who would benefit from adjunctive therapies to standard positive airway pressure (Stanchina et al., 2015).

By identifying patients who would be suitable for EERS therapy prior to standard CPAP failure, there is the potential for improved long-term compliance and acceptance of CPAP therapy. In the original EERS paper, we reported that 80% of patients who were treated had given up therapy; we had a long-term “salvage” rate of about 50% at long-term follow-up. This is particularly important as CPAP therapy has been demonstrated to resolve central events related to loop gain over time in select patients (Kuzniar et al., 2008; Javaheri et al., 2009), but this relies on the establishment of successful and compliant CPAP use. Thus, EERS use has three global goals—enabling healing in those whose high loop gain features will resolve by improving short-term tolerance, long-term benefits in those who have persistence of control instability, and salvage of those who have already failed therapy (i.e., providing a “second chance” at therapy). As hypoxia is a key driver of acquired increases in loop gain, it is expected that those who have substantial hypoxia are likely to improve over time, while those who have minimal hypoxia and possibly genetically determined abnormality will have long-term persistence. Heart failure is an example of a condition where some patients do not have severe hypoxia yet can have overt periodic breathing; many patients however are hypoxic, and the combination of hypoxic and non-hypoxic mechanisms can markedly elevate loop gain.

There are currently multiple methods for identifying underlying high loop gain as a marker for standard CPAP failure and/or an indication for EERS. Besides overt CSA or TE-CSA, multiple morphological features on diagnostic polysomnograms can be used to detect likely underlying elevated loop gain including NREM predominance of respiratory events with stabilization during REM sleep, increased NREM sleep stage transitional instability, short-cycle (<30 s) self-similar events, and “banding” oxygen desaturations visualized on pulse oximetry (Thomas et al., 2004). Mathematical formulas to measure loop gain have been utilized routinely in the research settings or secondary analysis of clinical trial data (Sands et al., 2011; Stanchina et al., 2015; Joosten et al., 2017; Li et al., 2019). The Phenotyping Using Polysomnography (PUP) method is a promising, potentially scalable method, of estimating endotypes from existing polysomnograms by using changes in estimated minute ventilation to determine metrics of respiratory drive (Finnson et al., 2021). Objective measurement of respiratory self-similarity (respiratory events with clone-like timing and morphology) also aids in risk-prediction (Oppersma et al., 2021). Residual events after several months of CPAP use also is a useful marker of a person who may need therapy targeting high loop gain, though mechanical effects of an oronasal mask (Genta et al., 2020), high leak or sleep fragmentation may all contribute (Ni and Thomas, 2023). Current device algorithms for detecting residual apneas on CPAP therapy have been shown to have significant errors in detection, particularly with regards to the presence of short cycle (<30 s) periodic breathing (Ni and Thomas, 2023).

## Patient safety and monitoring

In our extensive clinical experience with over 1,000 active patients and over 10,000 patient years of use, the use of EERS has been demonstrated to be safe and well tolerated in general OSA populations, in addition to patients with significant comorbidities including heart failure with reduced ejection fraction (unpublished). Initiation of (or conversion to) a non-vented mask and addition of 50 cc EERS may be considered as empiric treatment (i.e., without in-lab CPAP titration with ETCO_2_ monitoring) in patients without risk factors for hypoventilation (normal serum bicarbonate, normal pulmonary functions, body mass index ≤ 40 Kg/M^2^, ≤ 20 min with oxygen saturation under 90%, absence of disorders known to cause hypoventilation, absence of opiate or baclofen use). This approach to empiric therapy was necessitated by the COVID-19 pandemic, and used successfully.

## Biocalibration of CO_2_

After the conventional setup for the polysomnogram recording, we routinely perform a “CO_2_ biocalibration.” With a NV mask, mainstream ET CO_2_ sensor and no positive airway pressure, resting wake end-tidal CO_2_ is measured, followed by the change in CO_2_ with the addition of 50, 100 and 150 cc dead space. The sensor is placed at the mask outlet, and is able to capture the exhaled stream and provide a clear ETCO_2_ “plateau” in most instances. This measurement is quite sensitive to leak around the edges of the mask, and thus a very good fit is necessary for accurate tracking during sleep. While we originally had a set of recommendations for starting EERS volumes based on these values, we have shifted to using this maneuver as a safety check for unexpected hypercapnia. If the ETCO_2_ is ≥45 mm Hg, supervising on-call physician permission is required to use a non-vented mask. During titration, we tolerate a 5 mm Hg rise in ETCO_2_ but this threshold is virtually never reached except in opiate-induced CSA, as positive airway pressure provides a natural continuous washout. In over 5,000 sleep laboratory titrations with end-tidal CO_2_ monitoring, there has never been a single instance of induction of unexpected sleep hypercapnia in a patient with normal resting wake CO_2_. In patients undergoing in-lab non-vented CPAP titrations with EERS, continuous real-time ETCO_2_ monitoring is ideally utilized. With the non-vented configuration, end-tidal CO_2_ levels are generally readily attainable, including a clean plateau signal. However, reliable ETCO_2_ measurements can occasionally be limited by issues with mask fit and leak. Transcutaneous CO_2_ monitoring is a non-invasive method for obtaining accurate skin-surface oxygen and CO_2_ levels in the laboratory setting but is not critical if reliable ETCO_2_ levels can be obtained. The minimum general recommendation is to measure resting wake CO_2_ by any means including blood gas analysis prior to a non-vented titration.

## How to titrate EERS and positive airway pressure—practice points

In-lab titration of EERS and positive airway pressure relies upon careful understanding of the individual patient's underlying physiology, and often requires balancing control of obstruction through increased positive airway pressure against potential worsening of respiratory instability as mean airway pressures increase. As patients can differ drastically regarding the level of obstruction and underlying loop gain, as well as important parameters such as baseline sleep consolidation and arousal threshold, titration is therefore unique to each patient and should be performed in a physiologic rather than algorithmic fashion. That being said, there are a few key principles which should serve to guide successful titration of EERS.

Typically, titration is started with a non-vented mask setup with CPAP pressures at the lowest reasonable pressure to address obstruction (e.g., 6–8 cmH20). The decision to start the titration with additional EERS (50–150 cc's) is individualized and is dependent on (1) perceived level of underlying loop gain abnormality, (2) starting CO_2_ levels obtained prior to titration, and (3) the patient's tendency for prolonged sleep latency and poor sleep consolidation. This last point is important as adding additional EERS, as opposed to simply increased CPAP pressures, involves physically entering the room and making alterations to the patient's mask which will generate an arousal from sleep. No automated method to adjust EERS remotely currently exists. Regarding starting pressures, prior standard CPAP titrations can be useful to guide initial settings, with the understanding that standard titrations which do not address underlying loop gain often over-titrate in an attempt to stabilize breathing through increased pressures rather than CO_2_ modulation.

**Figure 3:**
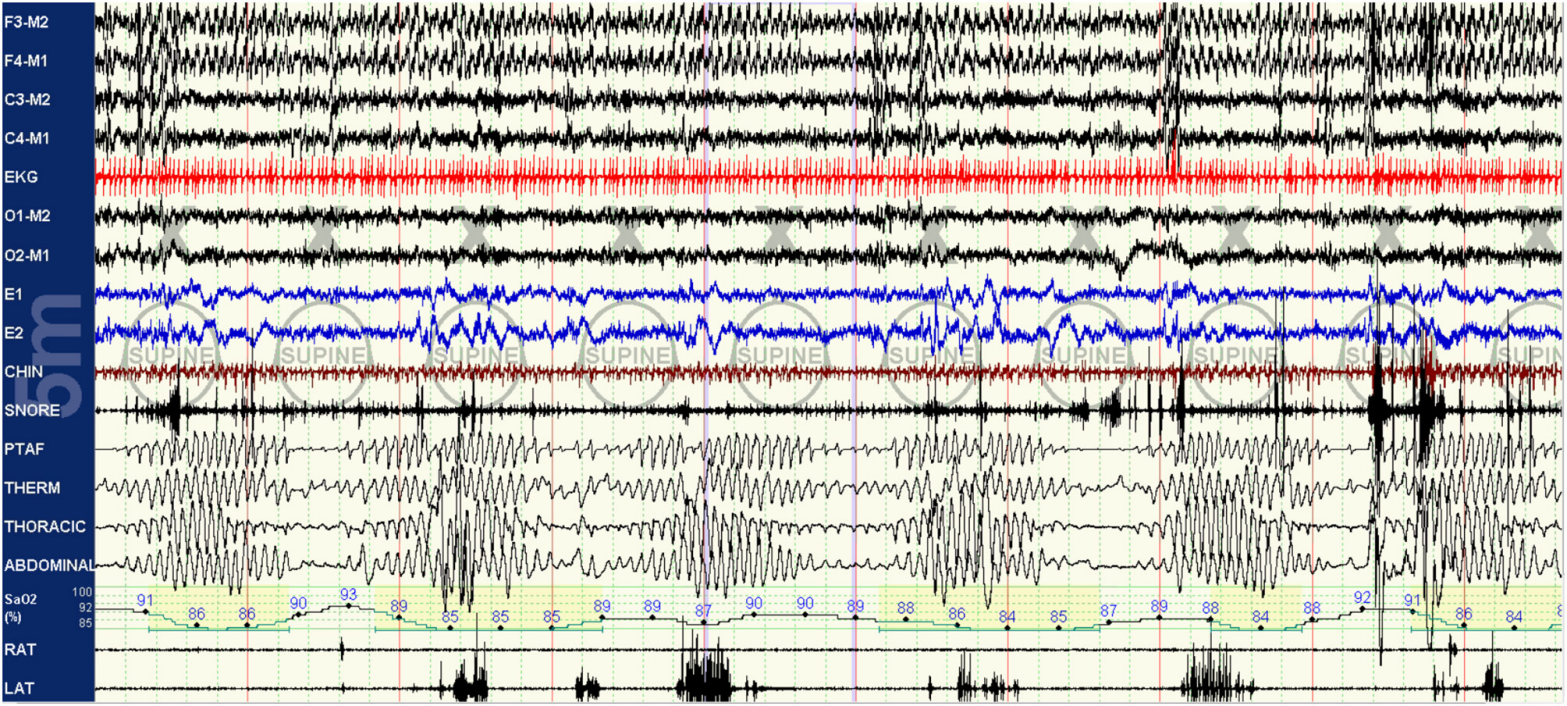
Baseline polysomnography: this baseline PSG of a patient with known sleep apnea demonstrates obstruction with clear high loop gain features including medium-cycle, self-similar waxing and waning respiratory events resulting in apneas and oxygen desaturations. Respiratory effort is present but minimal during apneic periods. The hypoxia nadir associated with an event actually occurs after the crescendo arm of respirations of the following cycle has concluded, indicative of a mixing delay.

**Figure 4:**
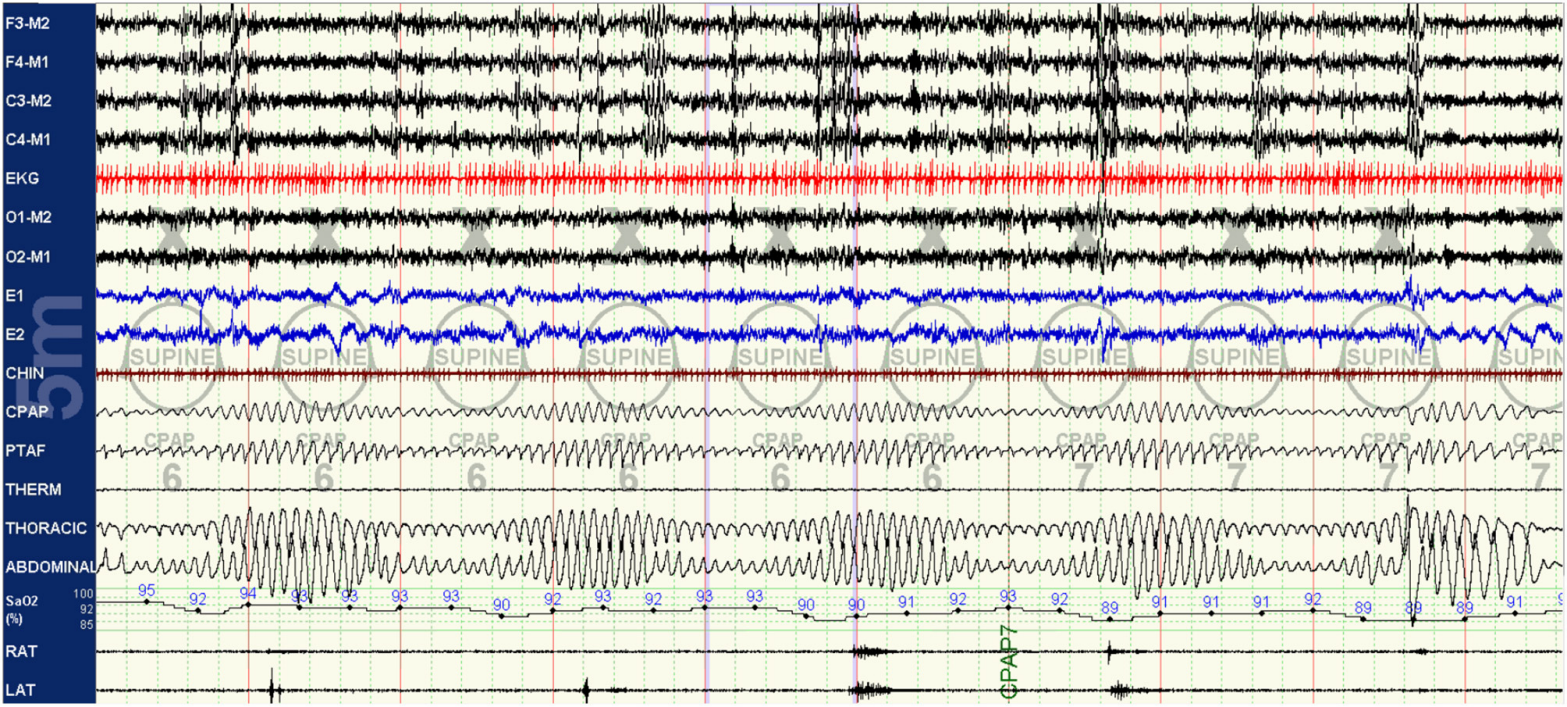
Standard CPAP titration: the patient was placed on standard, vented CPAP titration with no demonstrable improvement in breathing pattern with the use of positive airway pressure alone despite some improvement in obstructive physiology. The ongoing crescendo-decrescendo respiratory pattern and self-similar cycle suggest that the residual events are central rather than obstructive in nature despite the ongoing reduced respiratory effort. Despite guidance to score central hypopneas, the AASM scoring rules remains difficult for most sleep labs given the lack of use of esophageal balloon probes. Thus, identification of central hypopneas remains highly dependent on pattern recognition by the interpreting provider.

**Figure 5:**
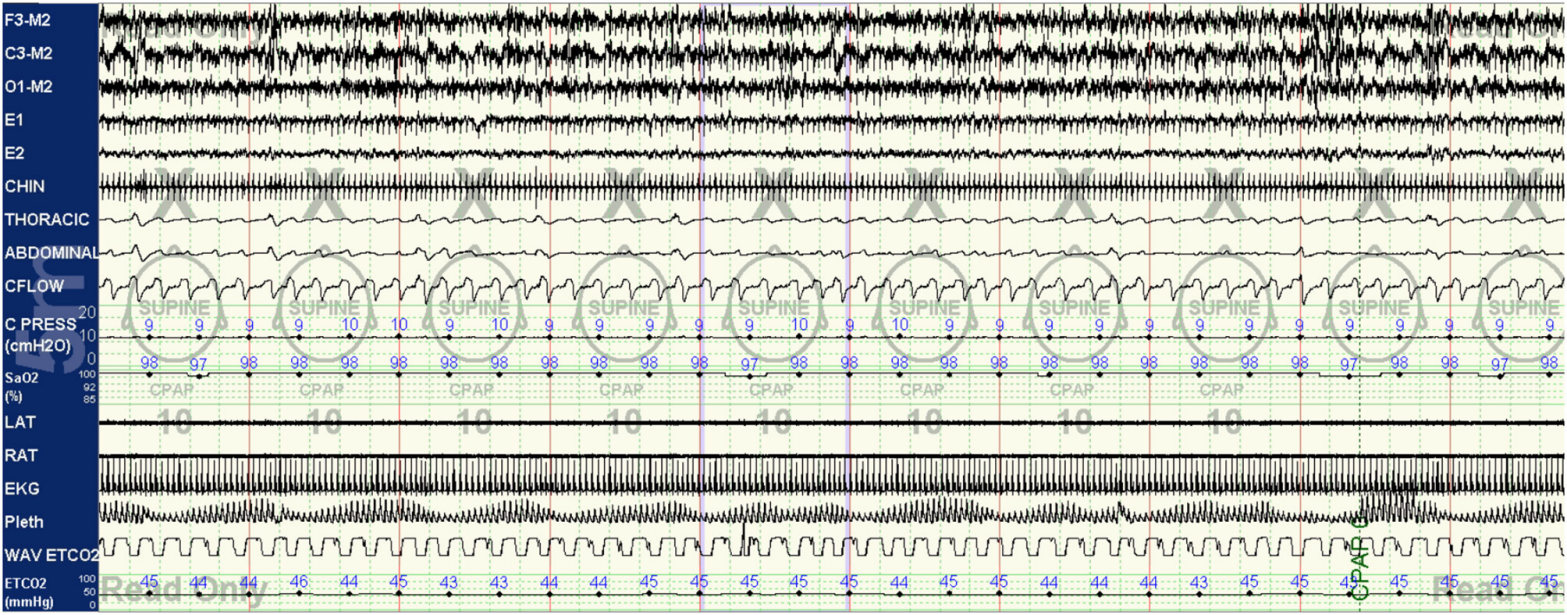
CPAP with EERS: the patient's CPAP titration is altered to include a non-vented mask and addition of EERS. With this change, breathing is immediately stabilized, with resolution of the crescendo-decrescendo respiratory effort of periodic breathing and normalization of oxygenation. The addition of EERS can allow successful application of higher CPAP pressures when needed to treat residual obstruction without augmenting instability. Of note, despite improvement in respiratory stability a cyclic pattern can still be observed in the plethysmogram signal, suggesting that the autonomic effects of the respiratory instability of high loop gain features are still not completely controlled. In some patients, cyclical arousals may persist despite stabilization of breathing. The serves as a testament to the complex multi-system integration of sleep physiology, with some components capable of demonstrating dissociated behavior.

As the titration progresses, pressures can be increased for clear flow-limitation and obstructive events, which typically predominate in REM sleep. Periodic (particularly short-cycle) obstructive events, especially in NREM sleep, should raise suspicion for high loop gain as a primary driver of the respiratory instability and aggressive up-titration of pressures should be avoided in favor of the addition of EERS if possible. It is often useful to intermittently and frequently review respiratory patterns in a 5–10 min window; this can help to visualize more subtle waxing and waning respiratory patterns seen as a result of high loop gain which can appear purely obstructive or even missed when viewing in a less compressed window (Figures 3–5).

Important aspects of the titration include stage/stability of sleep and body position. Both obstructive and high-loop gain physiology respiratory events are typically much more difficult to control in supine sleep. Therefore, an effort should be made to achieve respiratory stability in the supine position in both REM (typically predominantly obstructive requiring higher pressures) and NREM (typically predominantly loop-gain related and responsive to increased EERS). This balance is delicate and it is possible that respiratory control is unachievable in the supine position. If so, the titration can progress to avoidance of supine sleep utilizing the same principles, with non-supine sleep utilized as a primary therapeutic intervention.

## Proof of efficacy and safety

Dead space has some documented efficacy in hypocapnic CSA, though a prospective randomized trial of EERS is yet to be done. By using sub-tidal volume dead space with continuous wash-out with positive airway pressure, EERS enables an additive or even synergistic effect on respiratory control stability in sleep apnea care. EERS may be used with adaptive ventilation, a common practice in our center for patients that are difficult to control with either intervention alone. This combination typically reduces the range of pressure oscillations from the adaptive ventilator, improves tolerance, and reduces patient-ventilator desynchrony. Logically, EERS and standard bilevel ventilation would seem incompatible, but a rare patient may prefer the comfort of bilevel ventilation to CPAP. In a large-scale retrospective review of >200 patients with CPAP-refractory sleep apnea undergoing non-vented CPAP titration, control of disease was typically achieved when EERS volume was added to increase ETCO_2_ during sleep by just 1–2 mmHg above wake eupneic levels (mean ETCO_2_ 38.6 ± 2.9 mmHg at optimal therapy). Features of EERS titration vs. traditional CPAP titration from this retrospective review are summarized in Table 3 (Gilmartin et al., 2010). This study highlights the goal of EERS therapy in preventing nocturnal hypocapnia, rather than inducing hypercapnia, with EERS titrated for control of respiratory events rather than achievement of certain CO_2_ levels. Modest overall increases in ETCO_2_ with the addition of EERS additionally highlights the plausible mechanism of improved breathing control, which is likely secondary to reduction in plant gain and the amplitude of CO_2_ oscillations during sleep, rather than large increases in arterial CO_2_ levels.

**Table 3:** Retrospective analysis of 204 patients with CPAP refractory sleep apnea treated with EERS between 1/1/04 and 7/1/06.

** Measure**	**Diagnostic PSG**	**Standard Titration**	**EERS Titration**	***p*-value**
Sleep efficiency (%)	71.3 ± 18.2	66.9 ± 21.5	75.4 ± 14.9	<0.001
TST	222.4 ± 126.6	219.8 ± 105.2	308.5 ± 87.5	<0.001
Stage 1 (% TST)	21.6 ± 18	24 ± 19	20.3 ± 12.7	0.09
Stage 2 (% TST)	60.9 ± 16.8	56.7 ± 16.6	58.8 ± 13.1	0.05
Stage 3 (% TST)	5.8 ± 7.4	4.6 ± 6.5	5.5 ± 6.1	0.05
Stage 4 (% TST)	2.2 ± 6.3	1.2 ± 4.2	1.7 ± 4.2	0.10
REM sleep (% TST)	9.8 ± 9	13 ± 10.1	14 ± 9.5	<0.001
AHI 4% (/h of sleep)	36 ± 36.8	25.4 ± 59	4.1 ± 5.8	<0.001
RDI (/h of sleep)	69.8 ± 32.8	59.4 ± 33.9	30.7 ± 19.7	<0.001
CAI (/h of sleep)	3.8 ± 8.2	8.9 ± 11.1	1.5 ± 2.8	<0.001
Min O_2_	88.7 ± 8.1	88.5 ± 4.8	92.7 ± 4.5	<0.001
PLM index	6.1 ± 15.7	2 ± 4.8	15.2 ± 19.2	<0.001
16.54,15.5498pt **EERS titration finding**	**Value**
EtCO_2_ mean wake	38.1 ± 3.1 mm Hg
EtCO_2_ minimum when respiratory control achieved (minimum 50ml EERS)	38.6 ± 2.8 mm Hg
EtCO_2_ maximum for the study	42.1 ± 3 mm Hg
Throbbing headache on arousal	0
Headache attributable to mask straps	11 (5.4%)
Palpitations	0
Dyspnea	0

All patients' had in lab attended polysomnography for diagnosis, conventional CPAP titration, and further titration of EERS. Polysomnographic characteristics are displayed as well as CO_2_ levels and reported complications (Gilmartin et al., 2010).

AHI, apnea hypopnea index; CAI, central apnea index; EERS, enhanced expiratory rebreathing spacel; Et CO_2_, end tidal carbon dioxide; PLM, periodic limb movement; PSG, polysomnogram; RDI, respiratory disturbance index; TST, total sleep time.

Dead space has also been shown to efficacious in the treatment of CSA in patients with heart failure, with the addition of 400–600 cc of dead space alone resulting in improved sleep quality and respiratory stabilization, without detrimental effect on stroke volume or cardiac index as measured by transthoracic echocardiography, heart rate, or blood pressure (Khayat et al., 2003). Other data suggests that CO_2_ modulation via dead space is effective in significantly reducing AHI in a majority of OSA patients with a wide range of chemoreflex gains, with improved control over other interventions such as hyperoxia and transient isocapnia (Xie et al., 2013). In clinical practice, the main limitations are excessive leak (site or total volume), mask fit (tightness) and amplification of borderline claustrophobia. While these are not unique to the use of EERS, the latter is less leak tolerant.

## Patient barriers to use

The use of non-vented masking with EERS is highly reliant on an adequate mask seal, as significant leakage (even 20–30 lpm which is considered acceptable in standard CPAP application) may “wash out” rebreathing space and results in loss of breathing stability (Gilmartin et al., 2010), depending on the site of leak. Therefore, achieving adequate mask seal is paramount in successful therapy. Patients who demonstrate excessive leak should undergo mask fitting, with consideration of adjuncts such a chin strap or lip tape to prevent washout through the mouth.

A potential barrier to adequate mask seal is the EERS tubing itself; given the additional materials required to be added to the circuit, patients may need to alter their typical sleep position or utilize clips or other positional aids to ensure mask seal remains adequate for the duration of the night. Patients who are particularly sensitive to temperature and humidity fluctuations, including those with claustrophobia, may have more difficulty adjusting to the rebreathing space and comfort controls should be adjusted accordingly.

Finally, the dead space traps some moisture. Some patients may find that there is too much condensation in the rebreathing space. Adjusting humidification or even omitting humidification entirely are strategies to consider. The ambient humidity and temperature will also matter, as condensation (“rain out”) is more common in winter months.

## Pharmacologic adjuncts

In patients with significant respiratory instability, medications aimed at stabilizing breathing may be useful in conjunction with EERS. The most commonly utilized medication in this setting is acetazolamide, a carbonic anhydrase inhibitor initially used for the treatment of altitude sickness. The drugs shifts the CO_2_ response curve to the left and lowers the NREM sleep apneic threshold. Acetazolamide has been demonstrated to reduce respiratory loop gain by approximately 40% in patients with OSA via reduction in plant gain, and reducing the ventilatory response to arousal (Edwards et al., 2012, 2013). In one study of 236 patients with high loop gain sleep apnea, the addition of 125–250 mg of acetazolamide to standard CPAP therapy resulted in a reduction in breathing related arousal index, AHI3%/AHI4%, and RDI when compared to CPAP alone (Ni et al., 2023), and is generally safe and well-tolerated. Other pharmacologic adjuncts for the treatment of high loop gain sleep apnea include zonisamide (Eskandari et al., 2014), topiramate (Westwood et al., 2012), sulthiame (Hedner et al., 2022) (all carbonic anhydrase inhibitors) as well as buspirone (Maresh et al., 2020; Giannoni et al., 2021), which could be considered for patients in whom acetazolamide is poorly tolerated or contraindicated. Oxygen can always be considered an additional adjunct, as it directly reduces chemoreflex gain (Franklin et al., 1997; Sasayama et al., 2009; Yayan and Rasche, 2016).

## Considerations for home monitoring

In home settings, patients should be monitored for appropriate use of the EERS circuit. The increased complexity and need for after-market modification, combined with reduced familiarity of the technique with durable medical equipment (DME) providers means that there needs to be close collaboration with local DME companies. Practically this can increase risk for errors in application of the circuit for the patient. Compliance data is key to track with particular attention paid to markers of respiratory instability including residual central apneas and periodic breathing. However, positive airway pressure devices likely underestimate these patterns and manual review of the breath-by-breath waveform data can be particularly useful in EERS patients (Ni and Thomas, 2023). Given leak's ability to washout the effect of EERS this should be tracked and aggressively addressed on compliance data. Persistent optimal respiratory and symptom control despite substantial leak should raise the question of whether the EERS modification is still necessary and could trigger a trial return to a typical “vented” mask set up. A subset of patients with high loop gain, specifically those with substantial hypoxia, will have complete resolution of respiratory instability with successful therapy.

## Conclusions

Sleep apnea has multiple endotypes and a substantial minority of patients have high loop gain and/or low arousal threshold which predisposes to respiratory instability and central apneas. This is often triggered by relative hypocapnia. Central and complex sleep apnea remain difficult to control with existing positive airway pressure modalities. EERS represents an affordable, and relatively simple modification of existing positive airway therapy to modulate CO_2_ and minimize the hypocapnia that can trigger central apneas. Retrospective data over more than 15 years of clinical use Suggest high rates of success in patients previously intolerant of CPAP and an excellent safety profile. This technique should be avoided in patients with baseline hypercapnia. As the technique typically only generates at most a 1–2 mmHg increase in CO_2_ it is unlikely to evoke clinically significant hypercarbia or sympathoexcitation. In order to expand the use of EERS, multi-center, randomized control trials of EERS are desired. Further studies are also warranted to examine the combination of EERS with pharmacotherapy aimed at treatment of high loop gain.

## Author contributions

All authors listed have made a substantial, direct, and intellectual contribution to the work and approved it for publication.
